# First detection and genotyping of *Enterocytozoon bieneusi* in pet golden hamsters (*Mesocricetus auratus*) and Siberian hamsters (*Phodopus sungorus*) in China

**DOI:** 10.1051/parasite/2022018

**Published:** 2022-03-22

**Authors:** Chaochao Lv, Jingsong Wang, Chen Li, Min Zhang, Weifeng Qian

**Affiliations:** 1 College of Animal Science and Technology, Henan University of Science and Technology No. 263 Kaiyuan Road Luoyang 471003 China

**Keywords:** *Enterocytozoon bieneusi*, Golden hamsters, Siberian hamsters, Genotype, Zoonotic, China

## Abstract

*Enterocytozoon bieneusi*, a common opportunistic pathogen, has been detected in humans and a wide range of animals worldwide. However, no information on the prevalence and molecular characterization of *E. bieneusi* in hamsters is available worldwide. In this study, fecal specimens were collected from 175 golden hamsters and 175 Siberian hamsters purchased from pet shops in three provinces of China. The average infection rate of *E. bieneusi* was 12.0% (42/350), with 14.9% (26/175) in pet golden hamsters and 9.1% (16/175) in pet Siberian hamsters. Four genotypes were identified in pet golden hamsters, including three known genotypes (D, Henan-II, and SHW5) and one novel genotype (named Ebph1). Five genotypes were found in pet Siberian hamsters, including one known genotype (D) and four novel genotypes (named Ebph2 to Ebph5). Genotypes D and Ebph2 were the dominant genotype in pet golden hamsters (23/26, 88.5%) and Siberian hamsters (9/16, 56.3%), respectively. Phylogenetic analysis showed that the *E*. *bieneusi* isolates clustered into two groups: Group 1 (D, Henan-II, SHW5, and Ebph1) and Group 3 (Ebph2 to Ebph5). To the best of our knowledge, this is the first report of *E. bieneusi* infection in golden hamsters and Siberian hamsters worldwide. The identification of four genotypes belonging to Group 1 of high zoonotic potential suggests that pet hamsters especially golden hamsters can be potential sources of human microsporidiosis.

## Abstract (fr)

*Enterocytozoon bieneusi*, un agent pathogène opportuniste commun, a été détecté chez les humains et un large éventail d’animaux dans le monde. Cependant, aucune information sur la prévalence et la caractérisation moléculaire d’*E. bieneusi* chez les hamsters n’est disponible. Dans cette étude, des échantillons fécaux ont été prélevés sur 175 hamsters dorés et 175 hamsters sibériens achetés dans des animaleries de trois provinces de Chine. Le taux d’infection moyen d’*E. bieneusi* était de 12,0 % (42/350), avec 14,9 % (26/175) chez les hamsters dorés et 9,1 % (16/175) chez les hamsters sibériens. Quatre génotypes ont été identifiés chez les hamsters dorés, dont trois génotypes connus (D, Henan-II et SHW5) et un nouveau génotype (nommé Ebph1). Cinq génotypes ont été trouvés chez des hamsters sibériens, dont un génotype connu (D) et quatre nouveaux génotypes (nommés Ebph2 à Ebph5). Les génotypes D et Ebph2 étaient les génotypes dominants, respectivement chez les hamsters dorés (23/26, 88,5 %) et les hamsters sibériens (9/16, 56,3 %). L’analyse phylogénétique a montré que les isolats d’*E. bieneusi* se regroupaient en deux groupes : le groupe 1 (D, Henan-II, SHW5 et Ebph1) et le groupe 3 (Ebph2 à Ebph5). À notre connaissance, il s’agit du premier signalement d’infection par *E. bieneusi* chez des hamsters dorés et des hamsters de Sibérie dans le monde. L’identification de quatre génotypes appartenant au groupe 1, à fort potentiel zoonotique, suggère que les hamsters de compagnie, en particulier les hamsters dorés, peuvent être des sources potentielles de microsporidiose humaine.

## Introduction

*Enterocytozoon bieneusi* is a common microsporidian parasite that infects humans and a broad range of mammals and birds [14]. The parasite can cause asymptomatic infection or self-limiting diarrhea in immunocompetent hosts, and life-threatening diarrhea in immunocompromized individuals such as human immunodeficiency virus (HIV)-infected patients, organ transplant recipients, and cancer patients [7]. Hosts acquire infection usually through fecal–oral transmission of spores from infected humans and animals via direct contact or by ingestion of spore-contaminated food or water [14].

Sequence analysis based on the internal transcribed spacer (ITS) region of the rRNA gene has identified 11 major phylogenetic groups and more than 500 genotypes of *E. bieneusi* from humans and animals [14]. Groups 1 and 2, which are of major public health importance, contain most potential zoonotic genotypes; while other groups (Groups 3–11) appear to be more host-specific, with apparently limited public health importance [14]. Nearly 30 rodent species belonging to nine families (Castoridae, Caviidae, Chinchillidae, Cricetidae, Echimyidae, Hystricidae, Muridae, Sciuridae, and Spalacidae) have been reported as hosts of *E. bieneusi*, and approximately 100 *E. bieneusi* genotypes have been identified in rodents worldwide [4–6, 8, 11, 13, 16, 18, 19, 25–27, 30–33]. Among them, genotype D is the most common detected from rodents in previous studies [24, 27].

Hamsters are extremely popular small pets that are kept by both young children and adults worldwide. In China, the most common pet hamster species are golden hamster (*Mesocricetus auratus*), and two dwarf hamsters, namely Siberian hamster (*Phodopus sungorus*) and Campbell hamster (*Phodopus campbelli*). Pet rodents can carry a variety of zoonotic pathogens including viruses, bacteria, and parasites (such as *Cryptosporidium*, *Giardia*, and *E. bieneusi*) [17, 20, 27]; zoonotic transmission of *E. bieneusi* has occurred from domestic guinea pigs to a child in Peru [3]. However, no information is available on the occurrence and genetic characteristics of *E. bieneusi* in hamsters worldwide. The purpose of the present study was to determine the prevalence and zoonotic potential of *E. bieneusi* in these animals.

## Materials and methods

### Ethics statement

The research protocol was reviewed and approved by the Research Ethics Committee of Henan University of Science and Technology.

### Sample collection

From September 2018 to October 2019, a total of 350 fecal specimens were collected from the two most common species (*n* = 175 each) of pet hamsters purchased from seven pet shops in three cities, i.e., Luoyang (Henan Province), Weifang (Shandong Province), and Xuzhou (Jiangsu Province) in China (Table 1). The hamsters in pet shops were all pets offered for sale. From each pet shop, 25 golden hamsters (*Mesocricetus auratus*) and 25 Siberian hamsters (*Phodopus sungorus*) were obtained. Upon arrival at the laboratory, each animal was immediately placed into a separate clean plastic box for collection of fresh feces. Each animal was raised separately, and only a single sample was collected from each animal. All the specimens were stored at 4 °C prior to DNA extraction (within one week). Only 1–10-month-old pet hamsters were available in these pet shops. All pet hamsters examined in this study showed no clinical signs of disease at the time of sample collection, and information on region, age, and gender of these animals was recorded.

**Table 1:** Prevalence and genotypes of *Enterocytozoon bieneusi* in pet golden hamsters (*Mesocricetus auratus*) and Siberian hamsters (*Phodopus sungorus*) in China.

Host	Characteristics		No. of animals	No. positive (%)	Genotypes (no.)
Golden hamsters	Region	Luoyang, Henan	100	15 (15.0)	D (13), Henan-II (1), Ebph1 (1)
(*Mesocricetus auratus*)		Weifang, Shandong	50	6 (12.0)	D (6)
		Xuzhou, Jiangsu	25	5 (20.0)	D (4), SHW5 (1)
		Total	175	26 (14.9)	D (23), Henan-II (1), SHW5 (1), Ebph1 (1)
	Age (months)	1–3	122	20 (16.4)	D (18), Henan-II (1), Ebph1 (1)
		4–10	53	6 (11.3)	D (5), SHW5 (1)
	Gender	Male	96	15 (15.6)	D (14), Henan-II (1)
		Female	79	11 (13.9)	D (9), SHW5 (1), Ebph1 (1)
Siberian hamsters	Region	Luoyang, Henan	100	8 (8.0)	Ebph2 (5), D (2), Ebph3 (1)
(*Phodopus sungorus*)		Weifang, Shandong	50	5 (10.0)	Ebph2 (2), D (2), Ebph4 (1)
		Xuzhou, Jiangsu	25	3 (12.0)	Ebph2 (2), Ebph5 (1)
		Total	175	16 (9.1)	Ebph2 (9), D (4), Ebph3 (1), Ebph4 (1), Ebph5 (1)
	Age (months)	1–3	106	11 (10.4)	Ebph2 (6), D (3), Ebph4 (1), Ebph5 (1)
		4–10	69	5 (7.2)	Ebph2 (3), D (1), Ebph3 (1)
	Gender	Male	104	9 (8.7)	Ebph2 (4), D (3), Ebph3 (1), Ebph5 (1)
		Female	71	7 (9.9)	Ebph2 (5), D (1), Ebph4 (1)

### DNA extraction

Each fecal specimen was washed with distilled water by centrifugation at 3000 r·min^−1^ for 10 min. Genomic DNA was extracted from approximately 200 mg processed fecal samples using an E.Z.N.A. Stool DNA Kit (Omega Biotek Inc., Norcross, GA, USA), according to the procedure recommended by the manufacturer. The extracted DNA was stored at −20 °C before it was used for PCR amplification.

### Molecular detection

DNA from each specimen was tested for the presence of *E. bieneusi* by nested PCR targeting a ~390-bp fragment of the ITS region, as previously described [2]. The external primers were EBITS3 (5′–GGTCATAGGGATGAAGAG–3′) and EBITS4 (5′–TTCGAGTTCTTTCGCGCTC–3′), whereas the internal primers were EBITS1 (5′–GCTCTGAATATCTATGGCT–3′) and EBITS2.4 (5′–ATCGCCGACGGATCCAAGTG–3′). 2×EasyTaq^®^ PCR SuperMix (TransGen Biotech, Beijing, China) were used for PCR amplification. The thermal-cycling procedure was used as previously described [27]. Positive control (DNA of guinea pig-derived genotype S7) and negative control (distilled water) were included in each PCR analysis. Secondary PCR products were examined by agarose gel electrophoresis and visualized by staining with GelStain (TransGen Biotech, Beijing, China).

### Nucleotide sequencing and phylogenetic analysis

Two-directional sequencing of positive PCR products was done by General Biol (Anhui, China). The nucleotide sequences obtained were aligned with available sequences in GenBank, using ClustalX 2.1 (http://www.clustal.org/) and MegAlign, version 7.1 (a tool in the software DNAStar, https://www.dnastar.com/). Genotypes of *E. bieneusi* were determined and named based on ~243 bp of the ITS region, according to the established nomenclature system [21]. A neighbor-joining tree was generated using MEGA 7 software (http://www.megasoftware.net/). The evolutionary distances were computed using the maximum composite likelihood method, and the reliability of branches in the tree was assessed by bootstrap analysis using 1000 replicates.

### Statistical analysis

Chi-square analysis was performed to assess the correlation between the prevalence of *E. bieneusi* and the age, gender, and region of pet golden hamsters and Siberian hamsters using SPSS, version 17.0 (Statistical Package for the Social Sciences). A difference was considered statistically significant when the *p* value was <0.05.

### Nucleotide sequence accession numbers

Representative ITS nucleotide sequences of *E. bieneusi* obtained from pet golden hamsters and Siberian hamsters in this study have been deposited in GenBank under accession numbers OM427481–OM427489.

## Results and discussion

To our knowledge, this is the first report of *E. bieneusi* infection in pet hamsters. In the present study, 26 (14.9%) of 175 pet golden hamsters and 16 (9.1%) of 175 pet Siberian hamsters were positive for *E*. *bieneusi* by PCR, with an average infection rate of 12.0%. Compared with other pet rodents, the prevalence of *E. bieneusi* in pet hamsters was higher than pet chinchillas (3.6%), similar to pet fancy rats (11.2%), but lower than pet squirrels (16.7%), chipmunks (17.6%), and guinea pigs (20.2%) [5, 6, 19, 27]. The infection rates of *E. bieneusi* in pet rodents could be influenced by many factors, such as animal species, host health status, age distribution, management and living conditions, different geographical regions, and sample sizes.

The prevalence of *E. bieneusi* in pet golden hamsters was higher than that in pet Siberian hamsters, but the difference was not significant (*p* > 0.05). In both golden hamsters and Siberian hamsters, although the prevalences of *E. bieneusi* in younger animals were higher than those in older ones, the differences in the prevalence of *E. bieneusi* in both species between different region, age and gender groups were not significant (*p* > 0.05) (Table 1). This finding was in accordance with the observations in previous studies on pet red-bellied tree squirrels, fancy rats and guinea pigs in China [5, 27].

**Fig. 1:**
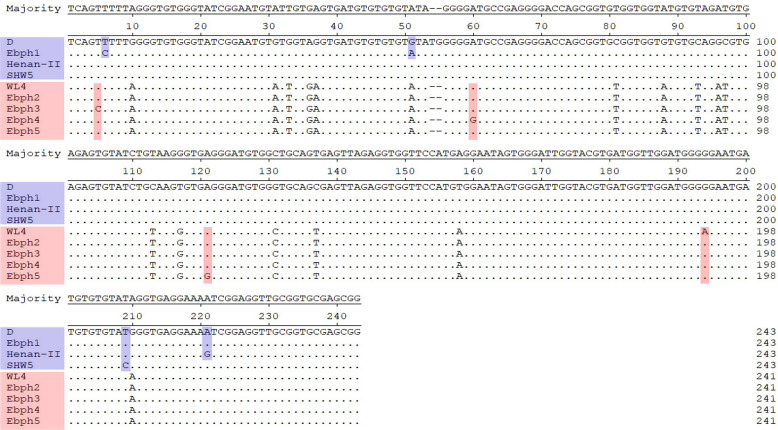
Nucleotide sequence diversity at the ITS region of *Enterocytozoon bieneusi* genotypes obtained in this study in comparison with related reference sequences.

**Fig. 2:**
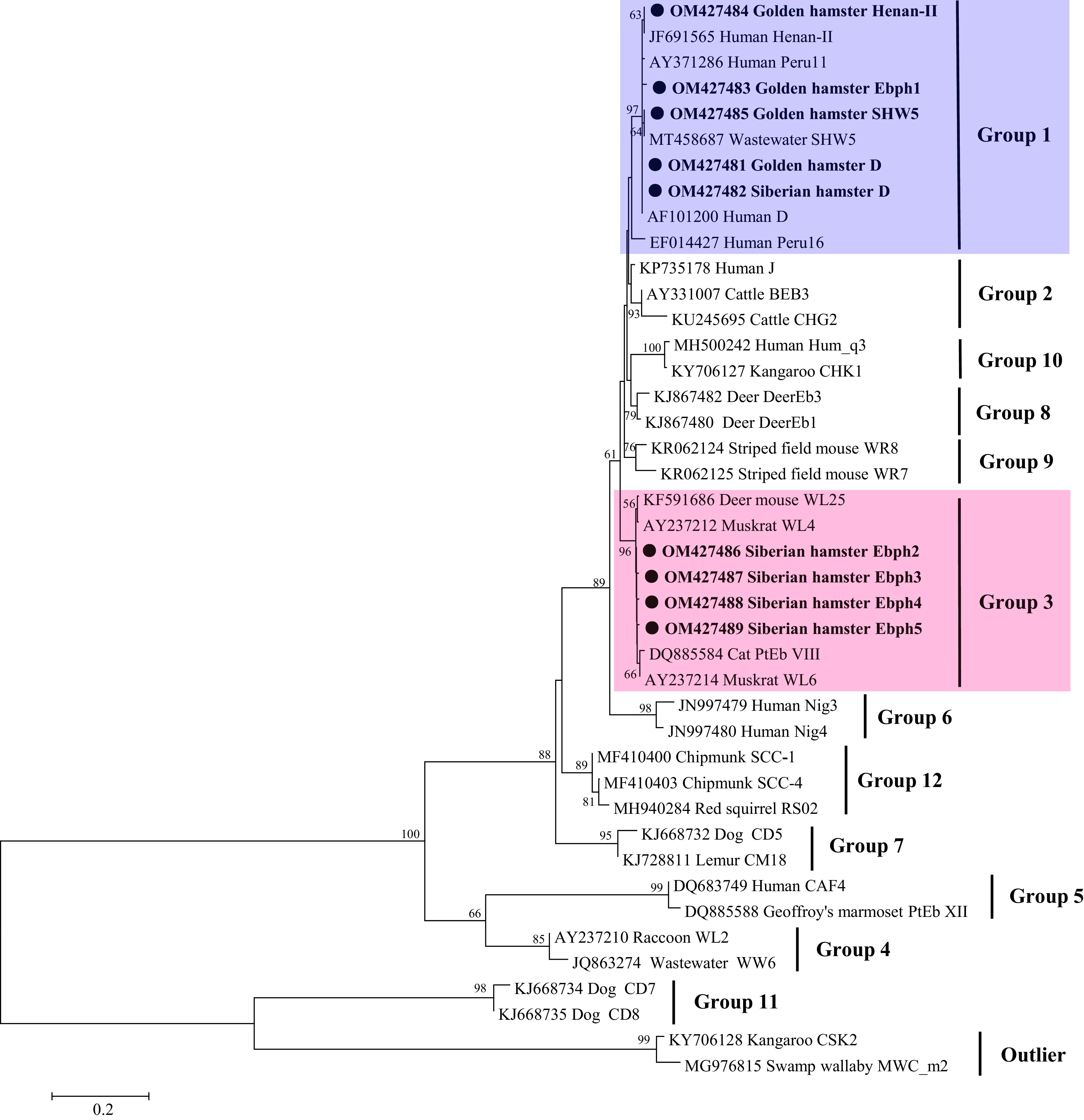
Phylogenetic relationships among the genotypes of *E. bieneusi* identified in this study and other known genotypes, as inferred by a neighbor-joining analysis of the ITS region. Bootstrap values above 50% from 1000 pseudoreplicates are shown. The genotypes identified in this study are indicated by closed circles.

Eight *E. bieneusi* genotypes, including three known genotypes (D, Henan-II, and SHW5) and five novel genotypes (named Ebph1–Ebph5), were identified by ITS sequence analysis in this study (Table 1). Genotype Ebph1 differed from D by two nucleotides. Ebph2 differed from WL4 (AY237212) by one nucleotide. Genotypes Ebph3 to Ebph5 differed from WL4 (AY237212) by two nucleotides each. Sequence differences among the five novel genotypes in the present study and their relatives (D or WL4) are shown in Figure 1. In the phylogenetic tree of the *E. bieneusi* ITS region, genotypes D, Henan-II, SHW5, and Ebph1 clustered into Group 1 of high zoonotic potential [13], and four novel genotypes Ebph2–Ebph5 clustered within the strong host-specific Group 3 (Fig. 2).

The *E. bieneusi* genotype distributions in the two hamster species were apparently different. In pet golden hamsters, genotype D (*n* = 23, 88.5%) was the predominant genotype, followed by Henan-II, SHW5, and Ebph1 (*n* = 1 each) (Table 1). For pet Siberian hamsters, the dominant genotype was Ebph2 (*n* = 9, 56.3%), followed by D (*n* = 4, 25.0%), and Ebph3–Ebph5 (*n* = 1 each) (Table 1). All the four genotypes in pet golden hamsters belonged to zoonotic Group 1; while expect for genotype D, the other four genotypes in pet Siberian hamsters were divided into host-specific Group 3. The difference is probably due to different host species. However, this observation is based on limited sampling sizes and regions, further studies on more samples collected from different regions should be conducted to understand the genetic diversity of *E. bieneusi* from hamsters in China.

In the present study, genotype D was the dominant genotype in pet golden hamsters, which is consistent with several previous reports from rodents such as pet and wild rats, pet red-bellied tree squirrels, pet red squirrels, domestic bamboo rats, and wild mice [4, 5, 13, 26, 27]. Genotype D is one of the most common zoonotic genotypes worldwide, and has been reported in several human cases in China [14, 29]. Genotype D has a broad range of animal hosts in China, such as non-human primates, livestock, companion animals, wildlife, and birds, as well as in wastewater [13, 14, 27]. In the present study, genotype D was identified in hamsters for the first time.

The genotype Henan-II was previously reported in an HIV-positive patient in Henan, China [28], and was found in a pet golden hamster for the first time. Genotype SHW5 was recently identified in wastewater samples from a hospital in Shanghai, China [10], and was found in a pet golden hamster in this study for the first time. Therefore, pet golden hamsters can be the source of human infection with the two genotypes, and more studies are needed to understand their host range and public health importance.

Six Group 3 genotypes have previously been reported, including WL6, WL22, WL23, WL25, PtEb VIII, and WL4 [13]. The first four genotypes are restricted to four rodent species in the USA [9, 23], and PtEb VIII has been found only in a cat in Portugal [15]. In contrast, genotype WL4 has been reported in a wider range of animal hosts, including wild rodents (squirrels, chipmunks, deer mice, and muskrats), and other mammals (raccoons, bears, otters, ermines, deer, and cottontails) in the USA [9, 22, 23], as well as in water samples in the USA, Tunisia, and China [1, 9, 12]. In the present study, the four novel genotypes (Ebph2–Ebph5) identified in pet Siberian hamsters, differed from WL4 by 1–2 nucleotides and clustered into Group 3. Genotypes Ebph2–Ebph5 may have a narrow host range and low or no public health importance.

## Conclusions

This is the first report of *E. bieneusi* infection in pet golden hamsters and Siberian hamsters. Three known genotypes and five novel genotypes were identified in this study, with zoonotic genotype D and host-specific genotype Ebph2 being the dominant genotype in pet golden hamsters and Siberian hamsters, respectively. The identification of four genotypes belonging to zoonotic Group 1 suggests that pet hamsters may be potential sources of *E. bieneusi* infection in humans. Therefore, pet owners, especially children, should be educated to take precautions to reduce the transmission risk.
